# Early gross motor performance is associated with concurrent prelinguistic and social development

**DOI:** 10.1038/s41390-025-03832-5

**Published:** 2025-01-17

**Authors:** Anastasia Gallen, Elisa Taylor, Juha Salmi, Leena Haataja, Sampsa Vanhatalo, Manu Airaksinen

**Affiliations:** 1 Department of Physiology, University of Helsinki, Helsinki, Finland; 2 Department of Clinical Neurophysiology, BABA Center, Pediatric Research Center, New Children’s Hospital and HUS Imaging, Helsinki University Hospital, Helsinki, Finland; 3 The Research Center for Psychology, Faculty of Education and Psychology, University of Oulu, Oulu, Finland; 4 Department of Pediatric Neurology, Children’s Hospital, Helsinki University Hospital and University of Helsinki, Helsinki, Finland

## Abstract

**Background:**

To study how early gross motor development links to concurrent prelinguistic and social development.

**Methods:**

We recruited a population-based longitudinal sample of 107 infants between 6 and 21 months of age. Gross motor performance was quantified using novel wearable technology for at-home recordings of infants’ spontaneous activity. The infants’ prelinguistic and social development was assessed in parallel with a standardized parental questionnaire (Infant Toddler Checklist). The developmental trajectories of motor, prelinguistic, and social performance were inspected longitudinally at individual level, and correlated to each other to measure the relative, age-adjusted advance in performance (z-scores).

**Results:**

Advanced gross motor maturation (higher z-score) links to more advanced prelinguistic development (*β* = 0.033, *p* = 0.016, *R*^*2*^ = 0.706) and social development (*β* = 0.038, *p* = 0.025*, R*^*2*^ = 0.600). When looking at specific gross motor skills, an increased amount of independent movement (crawling, standing, walking) links to more advanced prelinguistic and social abilities.

**Conclusion:**

We introduce a novel approach that measures individual level gross motor development longitudinally at high resolution from child’s spontaneous movements at home. This approach shows that age-adjusted relative advance in motor performance is linked to concurrent prelinguistic and social development, supporting the idea of developmental interaction across neurocognitive domains.

**Impact:**

- Early gross motor, prelinguistic, and social developments show trackable idiosyncratic trajectories.
- Maturity in gross motor performance links to concurrent prelinguistic and social development.
- Gross motor performance can be assessed reliably and objectively from infants’ spontaneous activity using unsupervised wearable recordings in their native environment, the homes.
- The present methodology with longitudinal quantitative assessments and age-adjusted modeling with z-scores introduce a potential paradigm shift to studying early neurodevelopment in the context of pediatric health, benchmarking of therapeutic interventions, and other developmental studies.

## Introduction

Infant’s early neurocognitive development is characterized by concurrently increasing repertoire of abilities in motor, cognitive, linguistic, and social domains.^1–3^ While these domains are often viewed separately, many recent studies indicate significant bidirectional links between them.^4–6^ For instance, infants’ ability to actively explore their surroundings is a prerequisite to learning of environmental affordances (functionally significant properties of the environment)^3^; or to engage in social interactions.^7,8^ Likewise, the onset of independent movement is followed by a significant increase in communicative skills at individual level.^9,10^

Traditionally, gross motor development is assessed by a clinician or parent via direct observations concerning reaching discrete milestones.^11,12^ Age at reaching a specific motor milestone can provide a simple measure, however, its assessment is subjective, and it ignores the natural continuum in increasing motor abilities.^13,14^ For instance, an infant who has just reached the ability to stand and walk is likely to exhibit this skill only sparsely compared to the more fluent, previously acquired skills (e.g., sitting and crawling) within the next weeks or months. A physiologically relevant motor assessment would thus benefit from a reliable and detailed quantification of infants’ all movements and postures. Such assessment became possible recently with the development and validation of a wearable method that provides detailed quantitative assessments using at-home recordings of infants’ spontaneous movement.^15–17^

Here, we aimed to study how early development of gross motor performance links to concurrent prelinguistic and social development, and in particular, how the different types of independent movements associate to the prelinguistic and social development. To this end, we used the novel wearable technology for quantifying motor performance during infants’ spontaneous activity at home,^15^ which was compared to a standardized parental questionnaire on infants’ prelinguistic and social development (Infant Toddler Checklist, ITC).^18^

## Methods

### Study overview

**Fig. 1:**
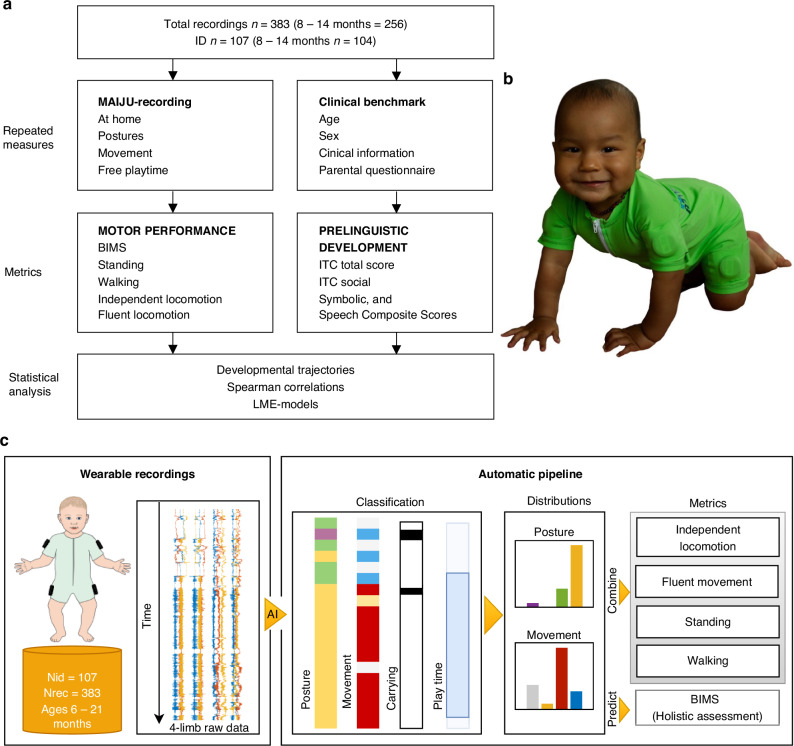
Study overview. **a** The flowchart depicts overview of the study protocol (see Fig. 2 for details of gross motor metrics). **b** MAIJU wearable used for quantifying infants’ motor performance at home. **c** Block diagram of the automatic analysis pipeline used to process the wearable recordings. First, AI-based classifiers are used to classify signal segments into parallel posture, movement, and carrying tracks (1.15-s resolution) and playtime segments (5-min resolution). The posture and movement distributions are obtained from the playtime segments and are used to obtain the final metrics.

The study design (Fig. 1) included repeated at-home wearable recordings of infants’ spontaneous playtime motility and parental questionnaires for longitudinal tracking of infant’s gross motor and prelinguistic development.

### Subjects

Participants from Finnish-speaking homes were recruited between 6 and 12 months of age for a longitudinal follow-up study that validates the wearable methods and characterizes infants’ gross motor development. In general, the longitudinal measurements were performed every two months until the age of 18 months. The study protocol was approved by the HUS Regional Committee on Medical Research Ethics (HUS/80/2021) and written informed consent was obtained from all caregivers by research personnel. The sample consisted of infants recruited via two channels (1) general advertising targeting typically developing infants with no known risk factors (*N* = 75) and (2) from the pediatric follow-up clinic due to developmental risk factors such as birth asphyxia or prematurity (*N* = 32). The following information was controlled in the analysis and found not to affect the present results: recruitment path, sex, parental education (ranging on a seven-point Likert-scale from elementary school to doctoral degree), and number of children in the family. The statistics for family background information are presented in the Supplementary Table S1.

### At home recordings of gross motor development

We used a multisensor wearable (“MAIJU”; Motor Assessment of Infants with a Jumpsuit, Fig. 1b) that is a whole-body garment equipped with four inertial measurement unit sensors (IMU), one in each limb.^19^ Each IMU records triaxial accelerometer and gyroscope data at 52 Hz (total 1248 samples per second) that was transmitted via Bluetooth to a nearby iOS mobile device using a custom-built iOS Maijulogger application (Kaasa GmbH, Düsseldorf, Germany). The suit is made from comfortable-to-wear swimsuit material, and the lateral proximal placement of the lightweight sensors within pockets make them very concealed from the point-of-view of the infant, thus unaffecting their behavior. Moreover, compared to video (or audio) recordings, the movement sensors have very low potential for capturing sensitive information from the home environment, which makes them very acceptable in terms of people’s sense of privacy.^20^ The suits were prepared ready for the recordings in our lab and then delivered to homes of the participants using local courier service. The parents dressed the suit on the infant and encouraged the infant to explore toys and move around for at least a total of an hour during the rest of the day. The suit was returned to the lab by the courier service, and the measured data was uploaded to a server “Babacloud” for a fully automated analysis pipeline (refs.^16,19^ see Fig. 1b. The analysis pipeline provides validated second-level detection of 7 posture and 9 movement categories. These postures and movements were analyzed from the time epochs that were automatically detected as infants’ spontaneous movement (“free play time”).^16^

### Metrics of gross motor development

#### Rationale

Previous literature has convincingly shown that the current posture and movement repertoire, and the overall gross motor maturity are essential for assessing infant motor development.^6^ Here we reasoned that the uncontrolled at-home wearable recordings provide the best possible ecological relevance to study infants’ spontaneous behavior, where any observer effects (e.g., presence of an unfamiliar adult; presence of a video camera) are minimized. Our recent works have shown that MAIJU recordings can quantify all these aspects of motor performance at human-equivalent accuracy, and the measures show clear individual level developmental trajectories.^15–17^

#### BIMS

Prior studies have shown that the overall gross motor maturity can be assessed efficiently by combining all the posture and movement detections during an at-home MAIJU recording.^16^ Such a combined measure is a scalar index (BIMS, BABA infant motor score) that ranges from 0 to 100 ^15^. With respect to motor performance, this range equals to progress from lying supine (near 0) to walking very fluently (near 100). Recently, BIMS was shown to correlate robustly with age, and it presents strongly individual differences in developmental trajectories.^16^ Therefore, BIMS provides an attractive index for assessing the relative advance in motor development by using a z-score that normalizes it for the given age (BIMS-z).

#### Independent movement

We quantified the incidence of different posture and movement patterns from the fully automated classifier outputs, which were combined for the present study into more intuitive and meaningful categories: Walking (‘standing’+‘elementary movement’ and ‘standing’+‘fluent movement’; i.e., spatial movement in standing posture), independent locomotion (‘elementary movement’, and ‘fluent movement’; i.e., spatial movement in any postural context), fluent locomotion (‘standing’+‘fluent movement’ and ‘crawl posture’+’fluent movement’; i.e., fluent walking and four-limb crawling). For a graphical illustration of these categories, see Fig. S1 in Supplementary material.

### Assessment of infant’s prelinguistic and social development

The infants’ prelinguistic and social development was assessed with the Infant Toddler Checklist.^18^ collected from the caregiver during each MAIJU measurement session. The ITC is a screening tool for communication skills in young children between the ages of 6 and 24 months, with high specificity and moderate sensitivity with regards to later neurodevelopmental outcomes.^21,22^ It consists of 25 questions for caregivers or professionals in regular contact with the child and yields a total score (ITC), as well as subscores including social composite (ITCsoc, items about emotion and eye gaze, communication gestures), speech composite (ITCspe, items about sounds and words), and symbolic composite scores (ITCsymb, items about understanding, object use). Here we used the previously translated Finnish language version. The ITC forms were delivered to homes of the participants using local courier service alongside the MAIJU suits and filled by parents at home.

### Statistical analysis

The statistical analysis was produced by R (R Core Team, 2013), R Studio (version 2024.04.1 + 748, R Studio Team, 2024), and MATLAB (version R2023, The MathWorks Inc., 2023).

To account for the confounding dependency due to chronological age in our measured variables, we constructed *z-scores* for both MAIJU (BIMS-z) and ITC related (ITC-z, ITCsoc-z) variables. The z-scores were calculated for participants with typical development based on monthly age-bins (6 to 21 months), for which raw mean and standard deviations were first obtained. The means and standard deviations were smoothed with 3-tap moving average filtering (except for the edge bins). The z-score for each recording was then obtained by searching for the closest age-group given the age at recording, and normalizing based on relevant smoothed mean and standard deviation.

Our data structure represents a hierarchical (non-independent) design, with repeated measures nested within-subjects. To analyze it more reliably, we utilized *linear mixed effects models* (LME; R Studio package lme4, function lmer). The id (individual infant) level was considered by adding an intercept for each individual. The constructed LME-models were compared with a log-likelihood ratio test (LR), Akaike’s information criteria (AIC), Bayesian information criteria (BIC), and pseudo-conditional *R*^*2*^ in rising order of complexity, and the most explanatory, yet parsimonious model, was chosen. As the models included both fixed and random effects, the models were fitted via maximum likelihood estimation.^23^

## Results

### Study subjects

Due to the requirements of the current study, we kept only the measurements that included a fully completed ITC questionnaire alongside a successful MAIJU recording with at least 60 min of successfully recorded infant playtime. For the overall motor performance with the BIMS index, there were a total of 107 subjects, from whom there were *N* = 383 available at-home measurements (age range 6–21 months; median number of measurements 4 range 1–8 per ID; average measurement interval 2.3 range 1–8 months; average 2.4 range 1–7 h playtime per recording). The age-restricted subset of the full dataset for quantifying independent movement between 8 and 14 months of age included a total of 255 sessions from 104 subjects. Higher parental education was associated with more advanced gross motor development (*F* = 5.12, *p* = 0.025), but it was not associated with child’s prelinguistic development (*F* = 2.17, *p* = 0.141). Number of children in the family was not associated with the child’s prelinguistic (*F* = 2.91, *p* = 0.090) or gross motor development (*F* = 0.04, *p* = 0.848).

### Individual trajectories for gross motor and social development

**Fig. 2:**
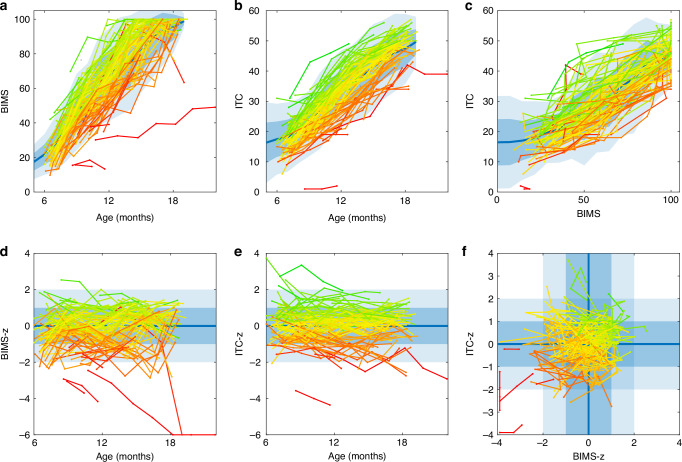
Individual trajectories of the motor and prelinguistic/social development. The upper row depicts the raw values of the holistic motor (BIMS; **a**) and prelinguistic/social development (ITC; **b**), as well as their mutual relationship (**c**). Each line depicts repeated measures of an individual infant (*N* = 107), and the lines have been colored according to the child’s mean z-score (from red (low z-score) to blue (high z-score). The lower row depicts the corresponding z-score trajectories for the motor (BIMS; **d**) and prelinguistic/social (ITC; **e**) development. The direct comparison between z-score trajectories (**f**) discloses a clear gradient of colors from the bottom-left (low z-score in both measures) to the top-right quartiles (high z-score in both).

The longitudinal tracking of the individual level motor performance (BIMS) and prelinguistic development (ITC) showed robust age-related trajectories (Fig. 2a, b). Indeed, the individual trajectories tended to follow their own ´growth channel´, or ´own percentiles´, which is robustly seen when color-coding the trajectories by their respective z-scores (Fig. 2a, b). Comparison of the z-score trajectories in BIMS and ITC allows assessing the age-adjusted relative advance at an individual level. Moreover, plotting the z-score trajectories against each other (Fig. 2f) reveals a developmental gradient where increasing BIMS-z (Fig. 2f, red) relates to an increasing ITC-z. Taken together, these longitudinal findings suggest that the relative advance in motor performance is likely linked to a comparable advance in the prelinguistic/social performance.

### Gross motor performance and prelinguistic and social development

#### Objective 1: Overall motor performance and prelinguistic and social development

**Fig. 3:**
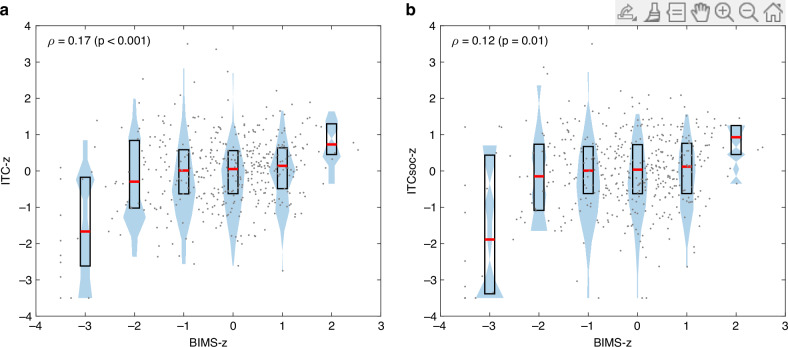
Prelinguistic and social development at different levels of age-adjusted motor performance. Increasing BIMS-z level (with corresponding median and interquartile range) relates to a robust increase in the ITC-z (**a**) and ITCsoc-z (**b**) levels, in particular at both extremes of the motor performance (BIMS-z).

**Fig. 4:**
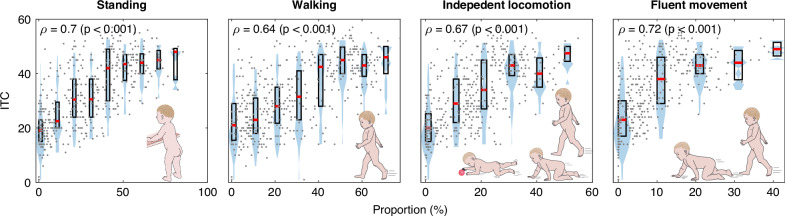
Violin plots (and corresponding medians and interquartile ranges) demonstrating the variations in independent movement categories vs. prelinguistic development (ITC-z). The associations between independent movement and prelinguistic skills were moderate (Spearman *r* = 0.35–0.47, *p* < 0.001).

As expected, all measures of motor performance and the prelinguistic skills show a strong mutual correlation (Fig. 3) that is due to an increasing performance with age in both domains. Therefore, a mutual, age-invariant relationship between the motor and prelinguistic domains was assessed from the respective z-scores (Fig. 4). The recruitment channel and child’s sex were sequentially added to the LME models as covariates to exclude possibility of any latent effects. The *motor only model* was significantly better than the intercept (id) only model (*χ*_*2*_ (379) = 5.76, *p* = 0.016), and overall gross motor performance (i.e., BIMS-z) was linked to the prelinguistic development, i.e., ICT-z, (main effect, *β* = 0.033, *p* = 0.016, *R*^*2*^ = 0.706, see Table 1, Fig. 3a). Sex or recruitment channel did not affect the link between BIMS and ITC (see Supplementary material, Tables S2 and S3).

**Table 1:** Results of the LME-models shown in parallel.

	Prelinguistic development		Social development	
	(ITC-z)		(ITCsoc-z)	
	*R*^*2*^ = 0.706		*R*^*2*^ = 0.600	
	*β*	*p*	*β*	*p*
*Intercept*	0.059	0.516	0.041	0.664
Motor performance (BIMS-z)	0.033	**0.016**	0.038	**0.025**
Random effects				
σ2 (residual)	0.323		0.521	
τ00 id [intercept]	0.767		0.770	

Prelinguistic (ITC-z) or social development (ITCsoc-z) as the outcome variable.

Likewise, we wanted to assess how infants’ overall motor performance (BIMS-z) is associated with their social development (ITCsoc-z) at 6 to 21 months of age (Fig. 3b). The *motor only model* was significantly better than the intercept (id) only model (*χ*_*2*_ (379) = 4.97, *p* = 0.026): The overall gross motor performance was strongly linked to social development (main effect, *β* = 0.038, *p* = 0.025, *R*^*2*^ = 0.600 Table 1). Sex or recruitment channel did not affect this link (see Supplementary material, Tables S4, S5). Motor development was not significantly related to symbolic or speech abilities (ITCsym-z or ITCspe-z).

### Independent movement and prelinguistic development

#### Objective 2: Independent movement, prelinguistic, and social development

We aimed to study, which aspects of the child’s motor skills at 8 to 14 months of age are associated with their prelinguistic and social development. To do this, we performed separate LME analyses with prelinguistic (ITC-z) and social development (ITCsoc-z) as the outcome. Measured independent movement skills (i.e., standing, walking, independent locomotion, fluent locomotion) were added to the models as fixed effects, and the nested within-subjects design was considered by adding an intercept for each individual id. These analyses excluded any other covariates.

The measured independent movement skills associated with prelinguistic and social development in a subset of 8- to 14-month-old infants (Fig. 4). Standing posture was linked to overall prelinguistic development (main effect, *F* = 8.15, *p* = 0.005), social development (main effect, *F* = 11.01, *p* = 0.001), and symbolic abilities (main effect, *F* = 5.86, *p* = 0.016). Likewise, regarding the movement categories, walking was associated to overall prelinguistic development (main effect, *F* = 8.78, *p* = 0.003), social development (main effect, *F* = 8.91, *p* = 0.003), and symbolic abilities (main effect, *F* = 5.87, *p* = 0.016). In other words, longer periods of standing or walking during the recording correlated to more advanced prelinguistic, social, and symbolic abilities. Accordingly, independent locomotion was linked to prelinguistic development (main effect, *F* = 7.01, *p* = 0.009), and symbolic abilities (main effect, *F* = 6.82, *p* = 0.010). Also, fluent locomotion was linked to prelinguistic development (main effect, *F* = 10.48, *p* = 0.001) and symbolic abilities (main effect, *F* = 9.28, *p* = 0.003), meaning that the more independent or more fluent movements were the more advanced was their prelinguistic or symbolic abilities. These results are presented in the Table 2.

**Table 2:** Type III analysis of variance tables with Satterthwaite’s method for the mixed effect models with random intercept for each subject.

		Prelinguistic development		Social development		Symbolic abilities	
		(ITC-z)		(ITCsoc-z)		(ITCsym-z)	
		*F*	*p*	*F*	*p*	*F*	*p*
Posture	Standing	8.15	**0.005**	11.01	**0.001**	5.86	**0.016**
Movement	Walking	8.78	**0.003**	8.91	**0.003**	5.87	**0.016**
	Independent Locomotion	7.01	**0.009**	2.94	0.088	6.82	**0.010**
	Fluent Locomotion	10.48	**0.001**	2.89	0.091	9.28	**0.003**

Fixed effects of standing, walking, independent locomotion, and fluent locomotion on prelinguistic (ITC-z) and social development (ITCsoc-z) and symbolic abilities (ITCsym-z). All variables denoted as z-scores. Total recorded *n* = 255, id *n* = 104.

## Discussion

The present findings show a significant link between several objective measures of gross motor performance and the concurrent prelinguistic and social development. A more advanced age-adjusted overall motor development, as well as a higher amount of independent movement were associated with a more advanced concurrent prelinguistic and social development. The findings are compatible with recent other studies suggesting relationships between motor and other developmental domains.^2,4,8,10,24^ However, the present work extends prior knowledge by reporting concurrent longitudinal tracking of these developmental dimensions, and by providing an objective and quantified assessment of infants’ motor performance in their native environments, at home. This made it possible to measure the full content of an infant’s spontaneous motor activity that is characterized by a natural mixture of different motor activities rather than reaching discrete milestones only.

Our findings and the previous literature support the idea that transition to independent locomotion or walking increases infants’ communicative activity and bids for social interaction.^2,7,10^ Increasing independent movement also deepens the verbal and physical communication with the parents.^25–27^ underscoring the effect of infant’s own motor abilities on the infant’s environmental exposure. Our present findings with accurate and objective quantifications suggest further that this link is significant for both the overall motor development level (BIMS) and the quantified amounts of independent moving in different ways. However, such statistical relationships in an observational study cannot disclose the dominant causal mechanisms, or direction of the effect. Nevertheless, our findings extend the current literature by demonstrating in a population-based sample that motor and communicative development are highly correlated at individual level over an extended period of time, and importantly, an age-corrected relative advance (z-score) in one domain relates to an advance in the other domain. It is intriguing to speculate that there may be joint underlying determinants, akin to general developmental phenotypes.^28,29^

Causal links between developmental domains could be further clarified by increasing the resolution of the present methodology in two ways: The precision of prelinguistic assessment could potentially be improved via a more objective formal expert’s assessment, such as ecological momentary protocol applied repeatedly and complemented by video recordings. Also, the motor assessment could be done more frequently using a more controlled environmental setting. However, these improvements would be notoriously labor intensive, and they would significantly compromise the ecological relevance that was achieved by the unsupervised, at-home MAIJU measurements and using parental questionnaires. For future studies, a particularly interesting approach would be to combine MAIJU recordings to parallel naturalistic long-form recordings of the speech environment obtained, e.g., with the LENA system.^30^ Earlier studies have addressed both motor and language development cross-sectionally whereas here we examine the development longitudinally. Moreover, the motor performance was quantified objectively in several intuitively explainable ways. Such study design disclosed stable individual trajectories in all developmental domains, which suggests that the overall finding was likely not compromised by the selected frequency of measurements, though a more frequent assessment could better assess possible differences in the temporal order of acquiring new skills.

### Strengths and limitations

A particular strength in our study is the use of wearable technology and the advanced, validated analysis algorithms, which can provide both holistic and detailed measures of the naturally variable motor behavior of an infant. Prior studies relying on binary milestone information are inherently ignoring the natural developmental continuum. Also, the inter- and intra-individual variation is challenging to capture with the neurodevelopmental measures.^31^ Here, we assessed the intra-individual variation in our measures by showing the strikingly robust individual developmental trajectories, and the statistical analyses were controlled for infant id, i.e., the extensive within-subjects variability. A possible caveat in our study is the use of caregiver questionnaire (ITC) for assessing prelinguistic development. While questionnaires are the standard approach in these studies, they always rely on subjective appraisal with a possible, inherent bias.^32^ Prior studies have shown, however, that the presently used ITC questionnaire shows a high specificity and at least moderate sensitivity with regards to later neurodevelopmental outcomes.^21,22,33^ We found no statistically significant association between prelinguistic performance and parental education, which may be seemingly conflicting with prior studies.^33^ However, such associations could be less clearly observed in the studied Finnish population of volunteering families with relatively homogenous educational and socioeconomic backgrounds than in more heterogenous samples. Also, the at-home measurement sessions with the MAIJU wearable were fully unsupervised: The parents were instructed to let the infants play freely during some segments of the day, but the exact characteristics of such sessions will always heavily depend on the home environment, infant-parent dynamics, etc. It is possible that some variability in the motor measures could be reduced by more control of the measurement session or with stricter caregiver instructions; however, it would come at the cost of compromising our aim to measure an infant’s spontaneous activity in their natural environment which we believe is best to represent an infant’s motor repertoire.

## Conclusions

The present study shows a significant link between different objective measures of gross motor performance and the concurrent prelinguistic and social development. A more advanced overall motor development and a higher amount of independent movement was associated with a more advanced prelinguistic and social development. This study adds to the growing evidence that different neurodevelopmental domains are linked together, such as the early motor development intertwines with prelinguistic and social skills. The present methodology may open a new way, even a paradigm shift, to model development of gross motor performance in a holistic, objective, and ecologically relevant way. Moreover, linking longitudinal trajectories of different developmental domains, and especially their age-adjusted levels (z-scores or percentiles from normative growth charts), hold promise for becoming efficient benchmarks in health care interventions, neurodevelopmental follow-up, as well as various kinds of developmental research.

## Supplementary information

SUPPLEMENTARY MATERIAL

## Data Availability

The datasets generated during and/or analysed during the current study are not publicly available due to privacy and ethical restrictions but are available from the corresponding author on reasonable request.
