# Supplementary material for: Early gross motor performance is associated with concurrent prelinguistic and social development
Source: Pediatr Res. 2025 Jan 17;98(3):1016–22. doi: 10.1038/s41390-025-03832-5 (PMC12507688; doi:10.1038/s41390-025-03832-5)
Supplement: Supplementary file 1 — SUPPLEMENTARY MATERIAL [file 41390_2025_3832_MOESM1_ESM.pdf]

SUPPLEMENTARY MATERIAL

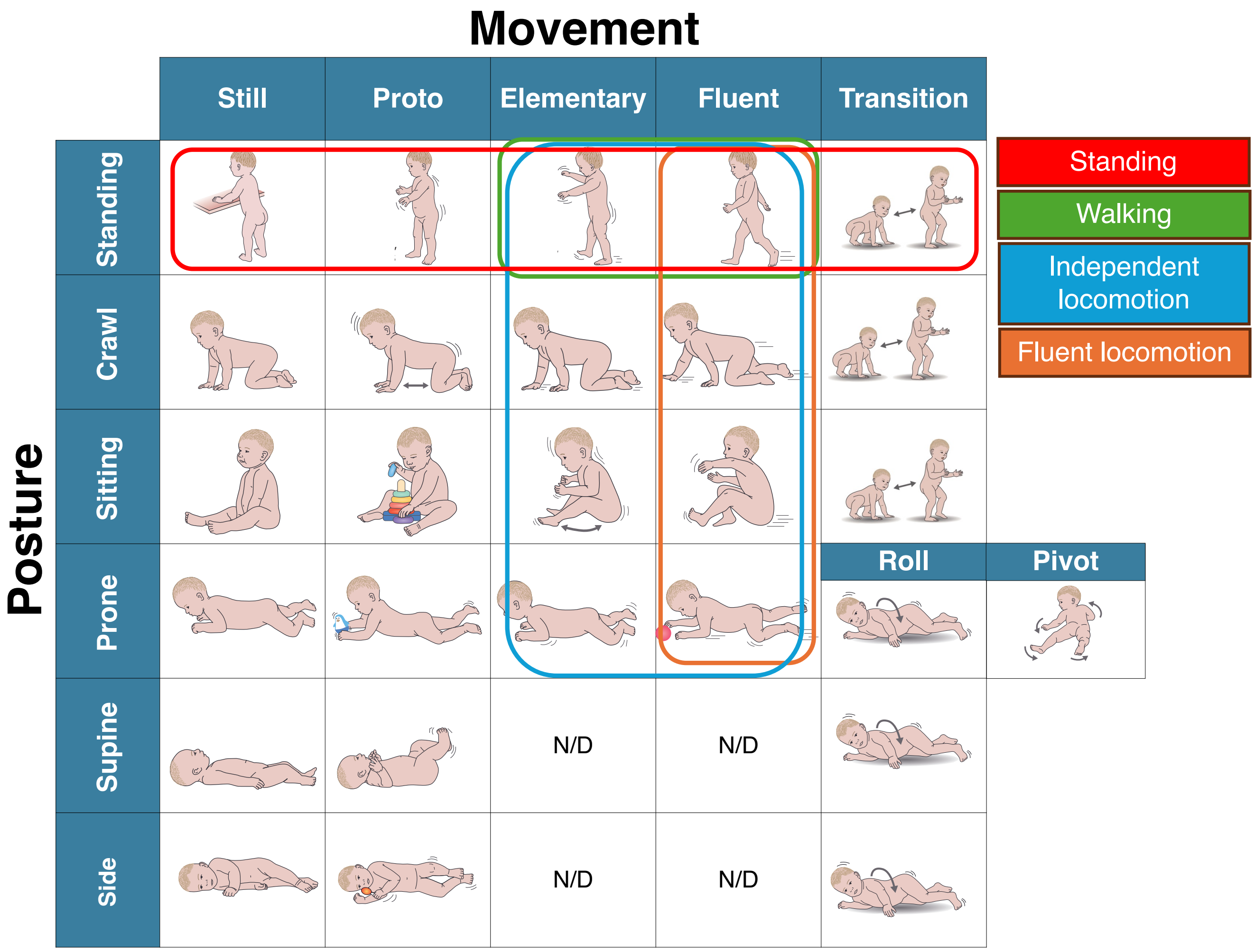

Figure S1. The Posture and movement categorization scheme used for the MAIJU analysis. The categories corresponding to the analyzed variables have been highlighted with colored boxes.

Table S1. Family background information. Respondents were mothers (n=101) and fathers (n=5, information missing n=1).

		n	Frequencies %	Mean	Median	Std. Dev.
**Parental education**				4.30	4	1.100
	1 = Comprehensive school	2	1.9%			
	2 = Upper secondary education	12	11.2%			
	3 = Vocational degree	0	0.0%			
	4 = Bachelor's degree	34	31.8%			
	5 = Master's degree	53	49.5%			
	6 = Doctoral degree	4	3.7%			
	Missing	2	1.9%			
**Family income level**				4.46	5	1.378
	1 = under 2000€	3	2.8%			
	2 = 2001€ – 3500€	4	3.7%			
	3 = 3501€ – 5000€	21	19.6%			
	4 = 5001€ – 6500€	23	21.5%			
	5 = 6501€ – 8000€	19	17.8%			
	6 = over 8000€	33	30.8%			
	Missing	4	3.7%			
**Parental age**				36.39	36	4.883
	Missing	1				
**Children in the family (n)**				1.64	1	1. 037
	1	61	57.0%			
	2	32	29.9%			
	3	7	6.5%			
	4	1	0.9%			
	5	3	2.8%			
	7	1	0.9%			
	Missing	2	1.9%			
**Relationship status**						
	Married	66	61.7%			
	Living together	36	33.6%			
	Unmarried	3	2.8%			
	Missing	1	1.9%			
**Perception of the child's development**						
	Typical	96	89.7%			
	Atypical	5	4.7%			
	Unable to tell	5	4.7%			
	Missing	1	0.9%			

Table S2. Model comparisons. Prelinguistic development (ITC total z-score) as the outcome.

	AIC	*BIC*	*log-Likelihood*	*LR χ_2 Difference Test*	*df*	*p*
The intercept-only model	896.89	908.73	-445.44			
Motor performance	893.13	908.92	-442.56	5.76	379	**0.016 ***
Motor + recruitment path	**893.25**	**912.99**	**-441.62**	**1.88**	**378**	**0.170**
Motor + recruitment path + sex	893.45	917.14	-440.73	1.80	377	0.180

Table S3. Models in rising order of complexity. Motor performance-model selected. Prelinguistic development (ITCtot-z) as the outcome variable, and the reference categories are well-child clinic recruitment path and girl sex. N rec total 383, N id 107.

	Motor performance ***R^2= 0.706***		Motor + recruitment path *R^2= 0.707*		Motor + recruitment path + sex *R^2= 0.708*	
	β	*p*	***β***	*p*	***β***	*p*
Intercept	0.059	0.516	0.140	0.197	0.244	0.067
Motor performance (BIMS-z)	0.033	**0.016***	0.032	**0.021***	0.032	**0.019***
Recruitment path			-0.272	0.172	-0.235	0.236
Sex					-0.243	0.182
**Random effects**						
**σ**2 (residual)	0.323		0.323		0.323	
**τ**00 id [intercept]	0.767		0.753		0.740	

Table S4. Model comparisons, social development (ITCsoc-z) as the outcome.

	AIC	*BIC*	*log-Likelihood*	*LR χ_2 Difference Test*	*df*	*p*
The intercept-only model	1035.90	1047.80	-515.00			
Motor performance	1033.00	1048.80	-512.49	4.97	379	**0.026 ***
Motor + recruitment path	1033.80	1053.50	-511.88	1.21	378	0.271
Motor + recruitment path + sex	1034.00	1057.70	-510.98	1.81	377	0.179

Table S5. Social development (ITCsoc-z) as the outcome variable. Models presented in rising order of complexity. Motor performance-model selected. The reference categories are well-child clinic recruitment path and girl sex. N rec total 383, N id 107.

	Motor performance ***R^2= 0.600***		Motor + recruitment path *R^2= 0.602*		Motor + recruitment path + sex *R^2= 0.604*	
	β	*p*	***β***	*p*	***β***	*p*
Intercept	0.041	0.664	0.108	0.337	0.217	0.118
Motor performance (BIMS-z)	0.038	**0.025***	0.037	**0.031***	0.038	**0.029***
Recruitment path			-0.227	0.273	-0.188	0.365
Sex					-0.254	0.181
**Random effects**						
**σ**2 (residual)	0.521		0.520		0.520	
**τ**00 id [intercept]	0.770		0.762		0.750	
